# Improving rice production sustainability by reducing water demand and greenhouse gas emissions with biodegradable films

**DOI:** 10.1038/srep39855

**Published:** 2017-01-05

**Authors:** Zhisheng Yao, Xunhua Zheng, Chunyan Liu, Shan Lin, Qiang Zuo, Klaus Butterbach-Bahl

**Affiliations:** 1 State Key Laboratory of Atmospheric Boundary Layer Physics and Atmospheric Chemistry, Institute of Atmospheric Physics, Chinese Academy of Sciences, Beijing 100029, P.R. China; 2 Institute for Meteorology and Climate Research, Atmospheric Environmental Research, Karlsruhe Institute of Technology, D-82467 Garmisch-Partenkirchen, Germany; 3 College of Earth Science, University of Chinese Academy of Sciences, Beijing 100049, P.R. China; 4 College of Resource and Environmental Science, China Agricultural University, Beijing 100193, P.R. China

## Abstract

In China, rice production is facing unprecedented challenges, including the increasing demand, looming water crisis and on-going climate change. Thus, producing more rice at lower environmental cost is required for future development, i.e., the use of less water and the production of fewer greenhouse gas (GHG) per unit of rice. Ground cover rice production systems (GCRPSs) could potentially address these concerns, although no studies have systematically and simultaneously evaluated the benefits of GCRPS regarding yields and considering water use and GHG emissions. This study reports the results of a 2-year study comparing conventional paddy and various GCRPS practices. Relative to conventional paddy, GCRPSs had greater rice yields and nitrogen use efficiencies (8.5% and 70%, respectively), required less irrigation (−64%) and resulted in less total CH_4_ and N_2_O emissions (−54%). On average, annual emission factors of N_2_O were 1.67% and 2.00% for conventional paddy and GCRPS, respectively. A cost-benefit analysis considering yields, GHG emissions, water demand and labor and mulching costs indicated GCRPSs are an environmentally and economically profitable technology. Furthermore, substituting the polyethylene film with a biodegradable film resulted in comparable benefits of yield and climate. Overall, GCRPSs, particularly with biodegradable films, provide a promising solution for farmers to secure or even increase yields while reducing the environmental footprint.

Agriculture is a major driver of global climate change^1^. The estimated source strength of agriculture is 5.1–6.1 Pg CO_2_-equivalents yr^−1^, contributing 10–12% to total global anthropogenic emissions^2^. Thus, producing more food to nourish a growing population while minimizing environmental costs, particularly by mitigating greenhouse gas (GHG) emissions, remains one of mankind’s greatest challenges^3^,^4^.

One of major agricultural commodities is rice, which is a major staple food for most people on earth and provides more calories for human consumption than any other cereal crop^5^. It is estimated that global rice production will need to increase by approximately 8–10 million Mg per year or by an annual yield of 1.2–1.5% in the coming decades to meet forecasted food needs^6^. Of particular concern is that increasing rice production corresponds with an increasing demand for water. Current estimates show that an average of 3000–5000 liters of water is needed for the production of one kilogram of rice, which is approximately 2–3 times greater than the water footprints of other cereal crops, such as wheat or maize^7^. Increasing urbanization and industrialization, however, are depleting water reserves and limiting the availability of irrigation water in many parts of the world, which particularly threatens the sustainability of irrigated rice systems^8^. Consequently, various studies are being conducted to explore water-saving technologies for rice production^9^,^10^,^11^,^12^. On the other hand, irrigated rice cultivation is a significant source of global CH_4_ emissions^13^. Also, high N_2_O emissions have been reported in fields with intermittent irrigation and midseason drainage or the excessive use or overuse of nitrogen fertilizer^14^,^15^. Recently, the IPCC^16^ estimated that rice production accounts for approximately 55% of the worldwide budget of GHG emissions from agricultural soils. Moreover, environmental problems associated with rice production will likely increase in the future because GHG emissions from rice fields and water scarcity (due to increasing frequency and severity of droughts) may increase as a result of climate change^17^,^18^. Consequently, rice systems are interrelated with food security, water scarcity and global climate change issues. However, currently available field scale and modeling studies have investigated these aspects of rice systems separately^19^,^20^. Thus, an integrated assessment considering water use, GHG emissions, rice productivity and the economic costs of different rice production systems as a basis for mitigation and adaptation strategies is missing.

Globally, China is one of the largest rice producing countries and is the second largest consumer of irrigation water^21^. Because China’s socioeconomic growth is expected to continue into the next decades, the associated increase in demand for water resources and rich foods can be reasonably projected^22^. Ground cover rice production systems (GCRPSs) have been developed by considering the diminishing availability of water for agriculture and the ever increasing demand for water in rice cultivation. For GCRPSs, the soil surface is covered with a thin plastic film and the soil moisture content is maintained near saturation with no standing water layer. This novel water-saving management practice has already been adopted in many provinces (more than 4 million hectares) of China^23^,^24^,^25^. Also, plastic mulching is commonly and increasingly used in East Asian countries other than China, such as Korea and Japan, and in Africa and the Middle East. The total area of arable land with plastic mulching is increasing annually by 15–20%^26^. Due to the increasing importance of GCRPSs in China, economic and environmental assessments are urgently needed. For example, GCRPSs with direct seeding have been reported to reduce water use, decrease rice yield, and increase total GHG (CH_4_ and N_2_O) emissions^27^,^28^. In contrast, several studies that have investigated GCRPSs with transplanted rice seedlings have indicated that this technology has resulted not only in irrigation water savings but has also ensured equal or greater rice yields^24^,^29^. However, the effects of these systems on N_2_O and CH_4_ emissions and economic costs have not been studied. Moreover, an obvious shortcoming of current GCRPSs is the use of a common polyethylene mulch film, which degrades extremely slowly and negatively affects the soil health and pollutes the environment. However, synthetic biodegradable polymers have become increasingly available and could provide a solution for overcoming this obstacle^30^,^31^.

In this study, we present the results from 2 years of continuous field measurements to assess water use, rice productivity and CH_4_ and N_2_O emissions in a Chinese subtropical rice-based cropping system under contrasting technology and management practices. The specific objectives of this study were to (i) determine the annual CH_4_ and N_2_O fluxes and their emission factors from different rice cultivation practices; (ii) assess the effects of GCRPSs with different mulching materials (polyethylene or biodegradable film) or different soil water statuses (near saturated or increased water stress) on the GHG emissions expressed on area- and yield-based scales; and (iii) identify a promising management option for maximizing irrigation water savings and yields while minimizing environmental impact.

## Results

### Seasonal and annual CH_4_ fluxes

**Figure 1:**
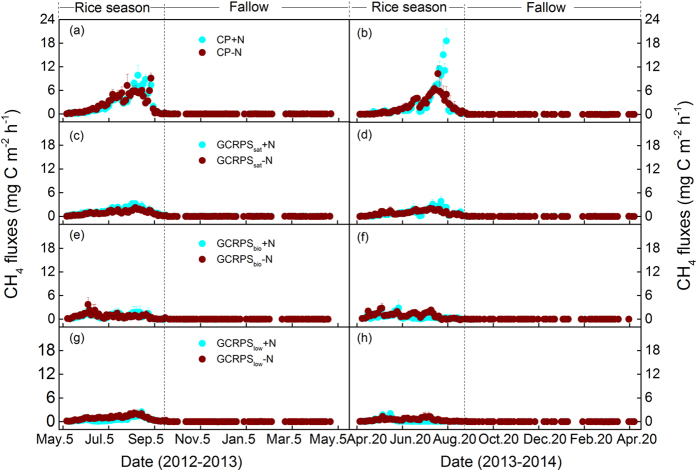
Seasonal variations of methane (CH_4_) fluxes for different rice cultivation practices fertilized using two nitrogen application rates during the period of 2012–2014. Vertical bars indicate the standard errors of three replicates. The legends in panels (a), (c), (e) and (g) also apply for the panels of the same row, respectively. Definitions of the treatment codes are referred to the footnotes of Table 1 and the text.

During the rice-growing seasons, the CH_4_ emissions were highly dependent on the water regime and soil moisture conditions (Fig. 1). In the CP system, the CH_4_ emissions continued to increase with rice growth, except for the midseason drainage in July, and peaked in mid-August (up to 9.0–18.5 mg C m^−2^ h^−1^) before decreasing thereafter. For the raised beds under GCRPSs, substantial CH_4_ emissions occurred but never exceeded 4.0 mg C m^−2^ h^−1^ and decreased to negligible when the soil was drained for harvesting. Compared with the raised beds, CH_4_ emissions from plant-free furrows were substantially lower (see Supplementary Table S2). Seasonal CH_4_ emissions varied across the rice-growing seasons and cultivation practices but were not significantly different between the N fertilized (+N) and unfertilized (−N) plots in each cultivation practice (Table 1). For the CP system, the seasonal CH_4_ emissions ranged from 71.3 to 85.5 kg C ha^−1^, averaging 80.2 kg C ha^−1^. Compared with the CP, the seasonal CH_4_ emissions were significantly reduced by 64% (P < 0.05), 73% (P < 0.05) and 81% (P < 0.05), on average, in the GCRPS_sat_, GCRPS_bio_ and GCRPS_low_ treatments, respectively. For all GCRPS treatments, the seasonal average CH_4_ emissions were lower in the GCRPS_low_ (15.5 kg C ha^−1^) than in the GCRPS_sat_ (29.1 kg C ha^−1^) and GCRPS_bio_ (21.8 kg C ha^−1^) treatments (see Supplementary Table S2). During the fallow periods, the soil CH_4_ uptake prevailed in all treatments. However, the soils occasionally served as a weak source of CH_4_. The cumulative CH_4_ fluxes across the fallow periods ranged from −0.25 to −1.02 kg C ha^−1^, without any significant treatment effects.

The average annual CH_4_ emissions from the different treatments ranged from 10.9 kg C ha^−1^ yr^−1^ (GCRPS_low_+N) to 83.5 kg C ha^−1^ yr^−1^ (CP+N) (Table 1). For all GCRPS treatments, the substitution of biodegradable film for polyethylene film (GCRPS_bio_) or increasing water stress (GCRPS_low_) did not significantly influenced the annual CH_4_ emissions compared to the GCRPS_sat_. However, plastic film mulching (GCRPS_sat_, GCRPS_bio_ and GCRPS_low_) reduced the average annual CH_4_ emissions by 73% compared to CP (P < 0.05).

### Seasonal and annual N_2_O emissions and their direct emission factors

**Figure 2:**
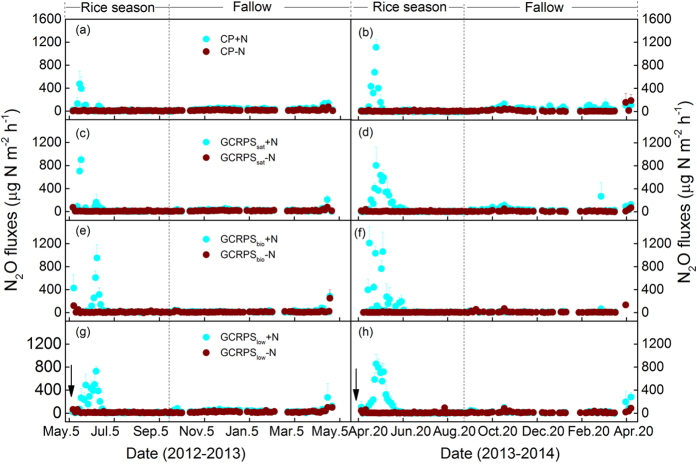
Seasonal variations of nitrous oxide (N_2_O) fluxes for different rice cultivation practices fertilized using two nitrogen application rates during the period of 2012–2014. Vertical bars indicate the standard errors of three replicates. The legends in panels (a), (c), (e) and (g) also apply for the panels of the same row, respectively. The arrows indicate the fertilization dates for each annual rice-fallow system. Definitions of the treatment codes are referred to the footnotes of Table 1 and the text.

The N_2_O emissions during the rice-growing seasons varied depending on the soil water status and N application rate, with peak emissions occurring within 1-2 months following fertilizer applications (Fig. 2). Although all urea-fertilized treatments showed comparable seasonal patterns, the magnitude and duration of peak emissions were greatly affected by the different cultivation practices. During the 2012 rice-growing season, the magnitude of N_2_O peak emissions in the CP+N plots (473 μg N m^−2^ h^−1^) was significantly lower than the magnitude in the GCRPS+N plots (727–951 μg N m^−2^ h^−1^) (P < 0.05). For the 2013 rice-growing season, the high emission (800–1200 μg N m^−2^ h^−1^) period following fertilization lasted approximately one month for the CP+N plots and approximately two months for the GCRPS+N plots. Relative to the CP+N treatment, therefore, the GCRPS+N treatments generated significantly greater N_2_O emissions during both growing seasons. Similar to the CH_4_ emissions, the N_2_O emissions from plant-free furrows were lower than those from the raised beds (see Supplementary Table S2). Across the rice-growing seasons, the lowest seasonal N_2_O emissions, with an average of 1.47 kg N ha^−1^, were observed for the CP+N plots, followed by the GCRPS_sat_+N (2.26 kg N ha^−1^), GCRPS_bio_+N (2.64 kg N ha^−1^) and GCRPS_low_ + N (2.98 kg N ha^−1^) plots, respectively.

Substantial N_2_O emissions also occurred during the fallow periods, particularly in April 2013 and April 2014 following heavy rainfall events. Thus, the application of N fertilizer not only results in higher N_2_O emissions during the growing season but also shows a significant legacy effect during the fallow period. Across the fallow periods, seasonal N_2_O emissions ranged from 0.37 kg N ha^−1^ for GCRPS_sat_-N to 2.29 kg N ha^−1^ for CP+N. Combining the total N_2_O emissions from the rice-growing and fallow periods, the CP, GCRPS_sat_, GCRPS_bio_ and GCRPS_low_ resulted in annual average N_2_O emissions of 0.96, 0.80, 0.78 and 1.27 kg N ha^−1^ yr^−1^ from the -N plots, and 3.47, 3.78, 3.40 and 4.71 kg N ha^−1^ yr^−1^ from the urea-fertilized (+N) plots. The differences between –N and +N plots were statistically significant (P < 0.05) (Table 1).

Averaged across two rice-growing seasons, the mean N_2_O EF_d_ values were 1.38%, 1.63% and 1.78% for GCRPS_sat_, GCRPS_bio_ and GCRPS_low_, respectively, and 0.88% for CP. The seasonal N_2_O EF_d_ for GCRPSs were significantly higher than those for CP (P < 0.05), while no significant differences were found among the GCRPS practices. When the emission factors were estimated based on the annual N_2_O emissions, the EF_d_ varied from 1.14% to 2.83% for all rice cultivation practices, with a mean of 2.00% for GCRPS and 1.67% for CP, respectively, with no significant difference between them (Table 1).

### Rice yields, nitrogen and irrigation water use efficiencies

Averaged across the 2 years and cultivation practices, the rice grain yields were significantly higher in the +N plots (6.88–7.57 Mg ha^−1^) than in the −N plots (5.91–6.37 Mg ha^−1^) (P < 0.05) (Table 2). Compared with the CP+N, the grain yields increased by 9.9% (P < 0.05), 5.4% and 10.1% (P < 0.05) on average in the GCRPS_sat_+N, GCRPS_bio_+N and GCRPS_low_+N plots, respectively, with a mean value of 8.5%. Among the GCRPS+N plots, the substitution of biodegradable film for polyethylene film (GCRPS_bio_) or further increasing the water stress (GCRPS_low_) did not significantly affected grain yield relative to the GCRPS_sat_.

Although the N contents in the rice plants at maturity were not significantly different among the treatments, the mean NUE across the two growing seasons was clearly higher for GCRPSs (25.5–36.8%) than CP (18.1%) (P < 0.05) (Table 2). Across the rice-growing seasons, the average irrigation water demand decreased as follows: CP (753 ± 47 mm) > GCRPS_bio_ (360 ± 17 mm) = GCRPS_sat_ (344 ± 28 mm) > GCRPS_low_ (119 ± 5 mm). The IWUE, which is an important indicator for the water use efficiency of a crop production system, was calculated by dividing the grain yield with the amount of irrigation water supplied. The values of IWUE were 0.91, 2.20, 2.01 and 6.36 kg grain m^−3^ water for CP+N, GCRPS_sat_+N, GCRPS_bio_+N and GCRPS_low_+N, respectively. Thus, compared with the CP treatment, the GCRPS treatments used 64% less irrigation water and the IWUE improved by 286% (P  <  0.05). Among the different GCRPS treatments, the GCRPS_low_ treatment was ideal because it required the lowest amount of irrigation water and had the highest IWUE.

### Total CH_4_ and N_2_O emissions

Similar to the area-scaled CH_4_ emissions, the yield-scaled growing season CH_4_ emissions were consistently lower for the GCRPSs than for the CP system (P < 0.05). Also, the trends and magnitudes of the effects of GCRPSs on the yield-scaled N_2_O emissions during the growing season relative to the CP were comparable to the effects of the area-scaled N_2_O emissions. Integrating CH_4_ and N_2_O emissions across growing seasons resulted in 115 to 692 kg CO_2_-eq Mg grain^−1^ season^−1^ (or 739 to 4672 kg CO_2_-eq ha^−1^ season^−1^) for all rice cultivation practices (see Supplementary Figs S6 and S7).

**Figure 3:**
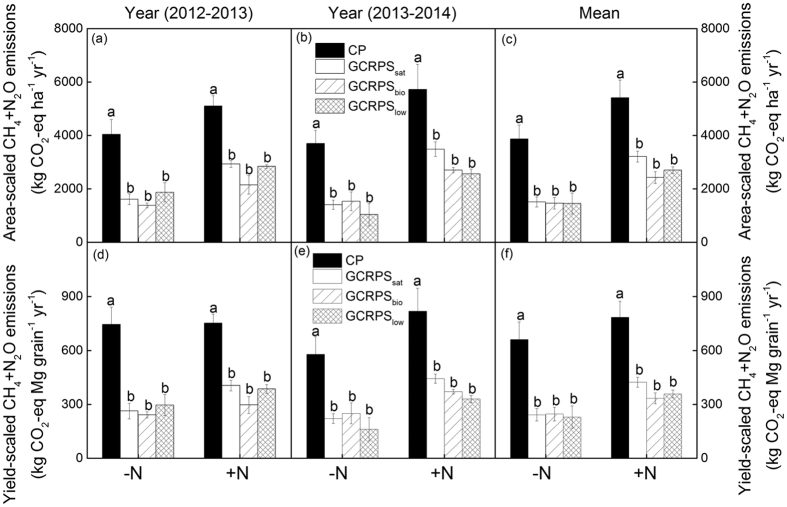
Annual area- and yield-scaled carbon dioxide (CO_2_) equivalents of methane (CH_4_) plus nitrous oxide (N_2_O) emissions for different rice cultivation practices fertilized using two nitrogen application rates during the period of 2012–2014. Mean represents the mean values of the two years. Vertical bars indicate the standard errors of three replicates in each rice cultivation practice. The area- and yield-scaled CO_2_ equivalents of CH_4_+N_2_O emissions for each N application rate followed by same letter are not significant at P < 0.05. Definitions of the treatment codes are referred to the footnotes of Table 1 and the text.

Averaged over the 2-year study, the annual CH_4_ and N_2_O emissions across all cultivation practices ranged from 229 to785 kg CO_2_-eq Mg grain^−1^ yr^−1^ (or from 1449 to 5410 kg CO_2_-eq ha^−1^ yr^−1^) (Fig. 3). Most emissions occurred during the rice-growing seasons, during which 65–93% of the total annual emissions occurred. Averaged across years and N application rates, the total CH_4_ and N_2_O emissions from GCRPSs compared to the CP system were reduced by 54% (P < 0.05), 60% (P < 0.05) and 59% (P < 0.05), when expressed on a grain yield basis (or by 49%, 58% and 55% when expressed on an area basis) in the GCRPS_sat_, GCRPS_bio_ and GCRPS_low_ treatments, respectively. Among the GCRPS practices, no significant differences were observed in the annual CH_4_ and N_2_O emissions.

## Discussion

Flooded rice systems are a significant source for anthropogenic GHG emissions because they emit substantial amounts of CH_4_^8^,^13^. Consequently, rice systems have higher total GHG emissions than any other major crop system, such as wheat or maize^32^, allowing for substantial mitigation. Our study shows that the introduction of GCRPSs significantly reduced the annual CH_4_ emissions, which were dominated by emissions during the rice-growing season (Table 1). The large CH_4_ mitigation potential of GCRPSs is mainly driven by the improved aeration of the topsoil because the soil water statuses (70–85% WFPS) under GCRPS (see Supplementary Figs S3, S4 and S5) prevent the development of strictly reducing conditions. Consequently this reduced topsoil methanogenesis and increased the CH_4_ consumption by methane-oxidizing bacteria^8^,^33^. Measurements of the redox potential (Eh), which are rarely performed in conjunction with WFPS measurements at the seasonal scale^4^, support this interpretation because the average value was significantly higher for GCRPSs (88–210 mV) than for the CP (27 mV) (see Supplementary Figs S2, S3, S4 and S5).

An increase in the N_2_O emissions was observed in the GCRPSs compared to the CP across the rice-growing seasons (Table 1), which confirmed the results of previous studies^27^,^34^. As also observed in other studies^35^,^36^,^37^, the N_2_O emissions were largely influenced by the soil temperature, WFPS, and mineral N concentrations. Thus, lower N_2_O emissions for the CP can be explained by the strongly reductive soil conditions, which hamper the microbial oxidation of NH_4_^+^ to NO_3_^−^ by nitrification. Such prevailing strong anaerobic conditions not only allow denitrification becoming substrate limited, but also support the complete reduction of oxidized mineral N compounds (NO_3_^−^, NO_2_^−^, NO and N_2_O) to the final denitrification end product N_2_^36^. Potter *et al*.^38^, Dobbie *et al*.^39^, and Weller *et al*.^8^ performed model simulations or field measurements and showed that the higher N_2_O fluxes generally occurred when the soil water contents were ≥60% WFPS, with peak emissions often occurring in a range of 70–90% WFPS. The WFPS in the GCRPSs generally ranged from 70–85%, which provided optimal environmental soil conditions for N_2_O emissions. Apart from the soil water status, the higher N_2_O emissions in the GCRPSs also resulted from the integrative effects of soil temperature and N fertilization. For example, the higher magnitude or longer duration N_2_O emission peaks in the GCRPSs relative to the CP were mainly observed during the first 1–2 months following fertilizer application (i.e., the period during which the plastic film used for GCRPSs resulted in the highest increases in topsoil temperatures (+2.2–3.7 °C) compared to the CP (see Supplementary Figs S2, S3, S4 and S5). Thus, the N_2_O emissions in the GCRPSs were stimulated by the high mineral N availability following urea application (see Supplementary Figs S2, S3, S4 and S5) and the optimal soil water content as well as increased soil temperature. All of these factors strongly stimulated soil microbial processes such as mineralization and coupled nitrification-denitrification, fueling N_2_O emissions^35^,^36^. On the other land, Liu *et al*.^25^ provided evidence from soil ^15^N isotope profiles that GCRPSs have a high potential to reduce NH_3_ volatilization and nitrate leaching. These N losses are both sources of indirect N_2_O emissions. Based on several studies investigating effects of plastic film mulching on NH_3_ volatilization and nitrate leaching from paddy fields, we estimated that due to the use of plastic film NH_3_ emissions are reduced by approx. 38% while nitrate leaching is reduced by approx. 21%^40^,^41^. Fertilizer N losses due to NH_3_ volatilization for the conventional paddy systems have been reported to be in the range of 9–42%, with a mean value of 25%^42^. As our present fields were fertilized with 150 kg N ha^−1^, in GCRPSs NH_3_ volatilization was thus likely reduced from 37.5 to 23.3 kg NH_3_-N ha^−1^. Likewise, fertilizer N losses due to nitrate leaching for conventional rice paddies have been estimated to be in the range of 2–10%, which depended on soil properties and water percolation rates^41^,^43^. Accordingly, for our field study nitrate leaching losses ranging from 3–15 kg NO_3_-N ha^−1^ (mean: 9 kg NO_3_-N ha^−1^) can be expected for the conventional paddy, while this value would be reduced to 7.1 kg NO_3_-N ha^−1^ for the GCRPSs. Using the IPCC default value for indirect N_2_O emissions due to NH_3_ volatilization (0.010) and nitrate leaching (0.0075)^44^, this translates to a reduction of indirect N_2_O emissions for GCRPS system by (37.5*0.38)*0.010 + (9*0.21)*0.0075 = 0.16 kg N_2_O-N ha^−1^ or 35% as compared to conventional paddy system. That is, indirect N_2_O emissions from GCRPSs are lower, while direct N_2_O emissions are higher as compared to the CP. So far, however, there are only a few studies reporting on indirect N_2_O emissions or considering both, i.e., direct and indirect N_2_O emissions from crop cultivation. It is evident that approaches to quantify indirect N_2_O emissions should be revisited and that indirect emissions should be included in estimates of N_2_O losses from different agroecosystems.

It should be noted that although our seasonal N_2_O measurements supported previous findings (e.g., GCRPS generally increased N_2_O emissions during the rice-growing season), we observed no significant differences in annual N_2_O emissions between the GCRPS and CP treatments (Table 1). This is because the N_2_O emissions from CP during the fallow period off-set the increased N_2_O emissions from the GCRPS at the beginning of the rice-growing season. Liu *et al*.^45^ has reported that water regime during the rice-growing season plays an important role on N_2_O emissions during the following upland period. They also observed that while N_2_O emissions from flooded fields during the rice-growing season were minor, substantially higher N_2_O emissions were occurring during the following fallow period, which were even higher than those from fields managed with water-saving technologies. These authors explained the higher N_2_O emissions during the fallow period in CP with priming effects on soil organic carbon mineralization following the switch from anaerobic to aerobic conditions^45^. This explanation was supported by the present finding that soil respiration (CO_2_) in CP was generally increased in the fallow season as compared to GCRPSs (see Supplementary Fig. S8). Therefore, one can only reinforce the general necessity for measurements spanning years that include fallow periods for obtaining the representative GHG emissions.

While several studies have evaluated the effects of GCRPS on yield and environmental parameters based on short-term measurements individually, to our knowledge, this is the first study in which multiple goals (water use, GHG emissions and rice yields) have been assessed in a single study. In this study, up to 753 m^3^ ha^−1^ of irrigation water was used for the CP treatment, which is typical for irrigated rice systems in Asia^5^. However, our study shows that implementing GCRPSs not only reduces irrigation water demand by 52–84%, but also increases the average rice yield by 8.5% compared with the CP system. Consequently, the IWUE nearly doubled (from 0.91 kg grain m^−3^ to 2.01–6.36 kg grain m^−3^) from the CP to the GCRPSs. Also, the mean NUE across the rice-growing seasons increased from 18.1% under CP to 25.5–36.8% under the GCRPSs (Table 2). The increased yields that occurred in the GCRPSs can be explained by the following factors. a) Long-term flooding of paddy soils can result in high concentrations of toxic reduction products, such as Fe^2+^, H_2_S and organic compounds, which seriously affect root growth^46^,^47^. Irrespective of N fertilization, improved aeration and increased soil Eh result in greater root biomass at soil depths of 0–10 cm and 10–20 cm and considerable root biomass at soil depths of 20–40 cm in GCRPSs relative to CP^25^. b) In accordance with the conclusions from previous studies^47^,^48^,^49^, greater root biomass and deeper rooting depths improve crop nutrient acquisition. c) Higher soil temperatures at the beginning of the growing season support crop development. d) Environmental nutrient losses due to leaching or NH_3_ volatilization are reduced in GCRPSs compared to CP^25^, increasing the availability of N for the crops and resulting in higher NUEs. This observation is an important finding and could be more attractive in adapting to water and food shortages due to climate change and population growth^18^,^50^.

In this study, the area and yield-scaled annual GHG (CH_4_ and N_2_O) emissions ranged from 1449 to 5410 kg CO_2_-eq ha^−1^ yr^−1^ and from 229 to 785 kg CO_2_-eq Mg grain^−1^ yr^−1^, respectively (Fig. 3), which fell within the range reported by Adviento-Borbe *et al*.^51^ for rice-fallow systems in the USA (658–7126 kg CO_2_-eq ha^−1^ yr^−1^ and 91–874 kg CO_2_-eq Mg grain^−1^ yr^−1^). As relatively low CH_4_ and N_2_O emissions can be obtained from rice systems through careful water management, the area and yield-scaled annual GHG emissions expressed in CO_2_ equivalents could be reduced by 54% and 58% on average, respectively, by converting rice paddies from CP into GCRPSs. However, it is noteworthy that in view of the longer atmospheric lifetime of N_2_O (121 years) as compared to CH_4_ (12.4 years) and the detrimental effect of N_2_O on the stratospheric O_3_ layer^16^, the current trend of increased N_2_O emissions from GCRPSs, specifically for GCRPS_low_ should be considered while developing mitigation strategies, although GCRPSs do reduce CH_4_ emissions substantially. On the other hand, a full evaluation of the mitigation potential of GCRPS must consider its influence on soil carbon stocks and effluxes. Although, in general, soil conditions with higher aerobic status and increased soil temperature would have stimulated organic matter mineralization and consequently decreasing soil organic C stocks, our recent and thorough regional scale evaluations have shown that the conversion from CP to GCRPS results in greater soil organic carbon (SOC) concentrations and storage^25^. This is mainly because GCRPS practices increase the above- and below-ground carbon inputs and improve the physical protection of soil organic matter against microbial degradation^25^. Overall, these results represent a win-win situation for agronomic and environmental goals because lower water use and GHG emissions can be obtained without compromising rice yields.

Further cost-benefit analysis suggests that GCRPS is a viable option for rice production from both environmental and economic points of view because of its monetary benefits associated with the GHG mitigation and because the water saving and yield increase outweigh the additional costs associated with labor, manufacture and purchasing the film (see Supplementary Table S3). Based on our economic assessment, the net benefits of implementing GCRPS_sat_, GCRPS_bio_ or GCRPS_low_ instead of using CP is in the range of $39.8–251.5 ha^−1^ yr^−1^, which is in order of GCRPS_low_ > GCRPS_sat_ > GCRPS_bio_ (see Supplementary Table S3). However, one major drawback of GCRPS_sat_ or GCRPS_low_ is their disposal, which could result in soil contamination and environmental pollution^26^. Although farmers manually collect the polyethylene plastic films in the GCRPS_sat_ after harvesting the rice plants, significant amounts of the plastic film remain in the field and accumulate in the soil profile, which creates a serious environmental problem^30^. Inspiringly, this study shows that non-decomposable polyethylene film can be successfully replaced with the biodegradable film Ecoflex^®^ (GCRPS_bio_). When using this type of film material, the positive effects of GCRPS_bio_ include the following: a) the mitigation of soil GHG emissions, b) a strong reduction in the demand for irrigation water and c) the stimulating effect on rice yields remain comparable to that observed for GCRPS_sat_ (Tables 1 and 2). Although revenues were higher for GCRPS_sat_ and GCRPS_low_ than for GCRPS_bio_, we recommend using GCRPS_bio_ because it avoids soil and landscape pollution resulting from the use of polyethylene plastic films^30^. This result is a novel finding that has not been reported in previous studies of GCRPS and will hopefully encourage the wider use of biodegradable films for rice production and other crop production systems. Due to the relatively high price for biodegradable films, however, governments should conceive effective policies and incentives such as providing subsidies for farmers to introduce the GCRPS_bio_ technology and management practice.

## Methods

### Study site and field treatments

From 2012–2014, field experiments were conducted on a farm (32°07′13″ N, 110°43′04″ E, 440 m above sea level) located at the Agricultural Bureau in Fangxian County, northwest of Hubei Province, China. The climate at the site is defined as northern subtropical monsoon^52^. The study region is a typical mountainous agricultural area, with one crop harvest per year. Paddy rice is the dominant crop, and fields remain fallow during the winter period. The topsoil has a silt loam texture and a pH (in water) of 6.0, and more soil properties were shown in the Supplementary Table S1. The daily precipitation and average air temperature during the experimental period are shown in Supplementary Fig. S1.

To assess management opportunities for reducing GHG emissions and water use while optimizing rice grain yield, four common rice cultivation practices and two nitrogen fertilizer application rates were tested. The resulting eight treatments were replicated three times using a randomized complete block design, i.e., the number of experimental plots was 24. Each plot was 9.0 m wide × 10.0 m long, and adjacent plots were separated by concrete ridges (40 cm width) and an impermeable film that was inserted into the soil to a depth of 90 cm.

The following four rice cultivation practices were used:

(1) Conventional paddy (CP), which is the local traditional paddy rice production system. In this system, plots were flooded between seedling transplantation until midseason, when the fields were drained for approximately 7 days. Following this period, fields were flooded again until they were drained approximately three weeks before harvesting the rice.

(2) Ground cover rice production system under nearly saturated soil water content conditions (GCRPS_sat_). These plots were separated into five raised beds (1.56 m width × 9.4 m length) surrounded by furrows (0.20 m width × 0.15 m depth) that were filled with water. However, no standing water was allowed in the raised beds. The raised beds were covered with regular polyethylene plastic film (1.70 m width and 0.005 mm thickness) with holes to allow for transplanting the rice seedlings.

(3) Ground cover rice production system with biodegradable films (GCRPS_bio_). In this treatment, water management was comparable to that described for (2) GCRPS_sat_, but Ecoflex^®^ (BASF, Germany) biodegradable film, which can be metabolized by soil microorganisms and is almost completely decomposed within a growing season, was used^31^.

(4) Ground cover rice production system with regular polyethylene film under lower soil water content conditions (GCRPS_low_) compared to (2) GCRPS_sat_. In this treatment, water management was identical to that described for (2) GCRPS_sat_ until the rice-regreening stage, which occurs approximately two weeks following transplanting. Following this initial period of near saturation, the soil water content was reduced to approximately 80% of that of the GCRPS_sat_ treatment by monitoring the soil moisture content.

For each rice cultivation practice, two nitrogen application rates were examined: (a) urea applied once before rice transplanting at a common rate of 150 kg N ha^−1^ (+N), and (b) no synthetic nitrogen fertilizer application (−N). To ensure that neither phosphate (P) nor potassium (K) limited crop growth, all plots received basal fertilization at application rates of 45 kg P_2_O_5_ ha^−1^ and 45 kg K_2_O ha^−1^.

The hybrid rice variety Yixiang 3728, which is a cultivar typically grown in the study region, was used for all of the tested rice cultivation practices. In 2012–2014, rice seedlings were transplanted on May 8, 2012, and April 28, 2013, and harvested on September 16, 2012, and September 10, 2013, respectively. After harvest, rice straw was completely removed and all fields were kept fallow in the winter season, which was in agreement with local practice.

### Measurements of CH_4_ and N_2_O fluxes

The fluxes of CH_4_ and N_2_O from the paddy rice-fallow systems were measured using the static vented chamber-based technique^21^. To account for the effects of micro-topography for plots managed as GCRPSs, two sizes of stainless steel frames, 65 cm × 90 cm × 15 cm and 20 cm × 30 cm × 20 cm (width × length × height), were inserted into the raised bed (accounting for 87% of the total area) and furrow (accounting for 13% of the total area) soils of each plot, respectively. For CP treatment, only one type of frame with dimensions of 65 cm × 90 cm × 15 cm was used in each replicated plot. The frames, that were positioned at least 1.5 m from the edges of the plots, were inserted into the soil to a depth of 15 cm, i.e., nearly reaching the compact plough pan layer. Some small holes were drilled in the frame below the soil line to allow for lateral water movement and root growth. Board walks were used to access the chambers and prevent soil disturbance. The planting density of rice crops between the inside and outside of the frame was similar. During the winter, all plots were drained and remained fallow, and the large chamber frames (i.e., 65 cm × 90 cm × 15 cm) remained in place to obtain flux measurements. The insulated chambers based on the type of frames (i.e., an area of 65 cm × 90 cm and a height of 100 cm and a 20 cm wide × 30 cm long × 30 cm tall) were used for gas sampling. For these chambers, two circulating fans were installed inside of the chamber headspace to facilitate mixing of chamber air and thus inhibiting the formation of gas concentration gradients. Also, a hole of 2 cm diameter was fitted in the top panel of sampling chambers, which could be left open when placing the chamber on the frame to prevent the build-up of over pressure within the chamber. Once the chamber was in place, this hole was connected to a pressure balance tube^21^.

Gas flux measurements were conducted three times per week during the experimental periods between 09:00 am and 11:00 am. Gas samples (40 ml) were taken from the chamber headspace at equal time intervals of 0, 10, 20, 30 and 40 min after covering by using polypropylene syringes fitted with three-way stopcocks. These samples were all analyzed within 6 hr of sampling by using a gas chromatograph (GC, Agilent 7890 A, Agilent Technologies, Santa Clara, CA, USA) equipped with a flame ionization detector for detecting CH_4_ at 200 °C and an electron capture detector for detecting N_2_O at 330 °C^21^. The CH_4_ and N_2_O fluxes were determined using linear or nonlinear regressions of gas concentrations versus the chamber closure time, as described in detail by Wang *et al*.^53^.

### Auxiliary measurements

During the experimental periods, we also measured the amounts of irrigation water, soil redox potentials (Eh), floodwater depths, soil volumetric water content, soil temperature, soil ammonium (NH_4_^+^) and nitrate (NO_3_^−^) concentrations, and aboveground biomasses (see Supplementary Information).

### Data processing and statistical analyses

The data were further processed for calculating total CH_4_ and N_2_O emissions, direct emission factor (EF_d_) of applied-N, irrigation water use efficiency (IWUE) and the fertilizer N-use efficiency (NUE) (see Supplementary Information).

To determine differences in GHG (CH_4_ and N_2_O) emissions among treatments during different observation periods (e.g., growing season) in the randomized complete block design, the SPSS 19.0 software (SPSS China, Beijing, China) was used with least significant difference tests with a P-value < 0.05. The cumulative CH_4_ and N_2_O emissions, their CO_2_ equivalents and grain yields due to main effects, such as rice cultivation practices, N fertilizer rates, year, blocking, rice cultivation practice × N fertilizer rate, year × rice cultivation practice × N fertilizer rate and block × rice cultivation practice as a random effect, were analyzed using Linear Mixed Models. The repeated measures ANOVA was used to test the effects of treatment on GHG emissions and environmental variables at a given period (e.g., peak emission period, growth stage).

## Additional Information

**How to cite this article**: Yao, Z. *et al*. Improving rice production sustainability by reducing water demand and greenhouse gas emissions with biodegradable films. *Sci. Rep.* **7**, 39855; doi: 10.1038/srep39855 (2017).

**Publisher's note:** Springer Nature remains neutral with regard to jurisdictional claims in published maps and institutional affiliations.

## Supplementary Material

Supplementary Information

## Figures and Tables

**Table 1:** Seasonal and annual cumulative fluxes of methane (CH_4_, in kg C ha^−1^) and nitrous oxide (N_2_O, in kg N ha^−1^) and the direct emission factor of applied nitrogen (EF_d_, in %) for the different rice cultivation practices fertilized using two nitrogen application rates during the rice-fallow systems of 2012–2014.

Year	Variable	CP†		GCRPS_sat_†		GCRPS_bio_†		GCRPS_low_†	
		−N	+N	−N	+N	−N	+N	−N	+N
2012–2013									
Rice season	Area weighted CH_4_*	81.4 ± 11.5 aA	85.5 ± 4.0 aA	25.3 ± 4.8 bA	36.6 ± 3.2 bA	22.1 ± 1.4 bA	21.9 ± 11.2 bcA	25.6 ± 10.0 bA	15.1 ± 6.7 cA
	Area weighted N_2_O*	0.12 ± 0.01 aA	0.98 ± 0.05 aB	0.22 ± 0.02 bA	1.53 ± 0.17 aB	0.27 ± 0.06 bA	1.74 ± 0.29 abB	0.38 ±0.04 cA	2.49 ± 0.69 bB
	EF_d_		0.57 ± 0.04 a		0.87 ± 0.11 a		0.98 ± 0.22 a		1.41 ± 0.47 a
Fallow	CH_4_	−0.81 ± 0.27	−0.70 ± 0.07	−0.84 ± 0.25	−0.70 ± 0.06	−0.25 ± 0.18	−0.83 ± 0.15	−0.69 ± 0.33	−0.56 ± 0.04
	N_2_O	0.70 ± 0.12	1.69 ± 0.44	0.85 ± 0.02	1.25 ± 0.40	0.55 ± 0.10	0.80 ± 0.15	1.20 ± 0.22	2.15 ± 0.29
Annual	CH_4_*	80.6 ± 11.4 aA	84.8 ± 4.0 aA	24.5 ± 4.6 bA	35.9 ± 3.3 bA	21.8 ± 1.4 bA	21.1 ± 11.1bcA	24.9 ± 10.2 bA	14.6 ± 6.7 cA
	N_2_O*	0.82 ± 0.12 aA	2.67 ± 0.49 aB	1.07 ± 0.02 aA	2.78 ± 0.54 aB	0.82 ± 0.05 aA	2.54 ± 0.36 aB	1.58 ± 0.24 bA	4.65 ± 0.62 bB
	EF_d_		1.23 ± 0.40 a		1.14 ± 0.24 a		1.14 ± 0.36 a		2.05 ± 0.44 a
2013–2014									
Rice season	Area weighted CH_4_*	71.3 ± 13.4 aA	82.7 ± 7.1 aA	26.3 ± 4.3 bA	28.1 ± 6.3 bA	26.7 ± 7.7 bA	16.3 ± 4.9 cA	13.7 ± 9.6 cA	7.71 ± 1.45 cA
	Area weighted N_2_O*	0.19 ± 0.05 abA	1.97 ± 0.16 aB	0.17 ± 0.03 bcA	3.00 ± 0.17 abB	0.11 ± 0.02 cA	3.54 ± 0.49 bB	0.25 ± 0.02 adA	3.46 ± 0.23 bB
	EF_d_		1.18 ± 0.11 a		1.89 ± 0.13 b		2.28 ± 0.33 b		2.14 ± 0.16 b
Fallow	CH_4_	−1.02 ± 0.17	−0.54 ± 0.07	−0.85 ± 0.10	−0.61 ± 0.13	−0.59 ± 0.03	−0.63 ± 0.07	−1.01 ± 0.09	−0.47 ± 0.10
	N_2_O	0.90 ± 0.37	2.29 ± 1.36	0.37 ± 0.07	1.78 ± 0.87	0.63 ± 0.02	0.71 ± 0.07	0.72 ± 0.07	1.30 ± 0.38
Annual	CH_4_*	70.3 ± 13.4 aA	82.2 ± 7.2 aA	25.4 ± 4.4 bA	27.5 ± 6.4 bA	26.1 ± 7.7 bA	15.6 ± 4.9 cA	12.7 ± 9.7 cA	7.24 ± 1.36 cA
	N_2_O*	1.09 ± 0.42 abA	4.26 ± 1.51 aB	0.54 ± 0.07 aA	4.78 ± 0.71 aB	0.74 ± 0.01 aA	4.25 ± 0.56 aB	0.97 ± 0.07 bA	4.77 ± 0.27 aB
	EF_d_		2.11 ± 1.09 a		2.83 ± 0.47 a		2.34 ± 0.38 a		2.53 ± 0.19 a
2012–2014‡									
Annual	CH_4_*	75.4 ± 7.5 aA	83.5 ± 3.6 aA	24.9 ± 1.1 bA	31.7 ± 4.1 bA	24.0 ± 3.3 bA	18.4 ± 5.6 bcA	18.8 ± 8.4 bA	10.9 ± 4.0 cA
	N_2_O*	0.96 ± 0.23 aA	3.47 ± 0.99 aB	0.80 ± 0.04 aA	3.78 ± 0.62 aB	0.78 ± 0.03 aA	3.40 ± 0.32 aB	1.27 ± 0.12 aA	4.71 ± 0.20 aB
	EF_d_		1.67 ± 0.75 a		1.98 ± 0.40 a		1.74 ±0.20 a		2.29 ± 0.10 a

The area weighted CH_4_ and N_2_O emissions in the GCRPS practices were calculated based on the areal extent of the raised bed (87%) and furrow (13%), and details are given in Table S1. ^†^The data shown are means ± standard errors (n = 3); CP, the conventional paddy rice production system with an initial flooding-midseason drainage-reflooding irrigation mode; GCRPS_sat_, the ground cover rice production system with polyethylene films, where the soil water content was held nearly saturated; GCRPS_bio_, the ground cover rice production system with biodegradable films, where water was managed the same as in the GCRPS_sat_ treatment; GCRPS_low_, the ground cover rice production system with the same covering film as the GCRPS_sat_ and with near saturation until the rice-regreening stage and at approximately 80% of the GCRPS_sat_ management for the reminder of the season; −N, no synthetic nitrogen fertilizer application; +N, a local common application rate of 150 kg N ha^−1^. *CH_4_ and N_2_O emissions within each row followed by the same lowercase letter are not significantly different among the rice cultivation practices under each N application rate at the P < 0.05 level, and those followed by the same capital letter are not significantly different between unfertilized and fertilized treatments under each rice cultivation practice at the P < 0.05 level. ^‡^Mean values of the two annual rice-fallow systems.

**Table 2:** The characteristics of the grain (in Mg ha^−1^) and straw (in Mg ha^−1^) yields and N uptake (in kg N ha^−1^) of aboveground biomass (i.e., grain+straw) as well as the estimated nitrogen use efficiency (NUE, in %) for the different rice cultivation practices fertilized using two nitrogen application rates during the rice-growing seasons of 2012 and 2013.

Rice season	Variable	CP^†^		GCRPS_sat_^†^		GCRPS_bio_^†^		GCRPS_low_^†^	
		−N	+N	−N	+N	−N	+N	−N	+N
2012	Grain yield*	5.42 ± 0.20 aA	6.78 ± 0.16 aB	6.19 ± 0.24 bcA	7.26 ± 0.25 aB	5.70 ± 0.06 abA	7.23 ± 0.07 aB	6.33 ± 0.14 cA	7.39 ± 0.37 aB
	Straw yield*	5.74 ± 0.26 aA	7.61 ± 0.45 aB	6.18 ± 0.32 aA	8.35 ± 0.78abB	5.89 ± 0.10 aA	9.45 ± 0.16 bB	6.93 ± 0.27 bA	9.14 ± 0.39 bB
	N uptake*	84.8 ± 5.7 aA	110 ± 10.2 aB	88.7 ± 5.1 aA	120 ± 2.1 aB	84.3 ± 4.8 aA	123 ± 5.3 aB	93.9 ± 3.5 aA	128 ± 6.0 aB
	NUE		17.1 a		21.1 a		25.7 a		22.4 a
2013	Grain yield*	6.47 ± 0.26 aA	6.98 ± 0.05 aB	6.36 ± 0.17 aA	7.86 ± 0.34 bB	6.11 ± 0.17 aA	7.27 ± 0.02 acB	6.40 ± 0.09 aA	7.76 ± 0.09 bcB
	Straw yield*	7.16 ± 0.28 aA	7.93 ± 0.07 aB	6.62 ± 0.26 aA	9.26 ± 0.29 bB	6.37 ± 0.20 aA	10.2 ± 0.80 bB	6.65 ± 0.22 aA	8.86 ± 0.29 bB
	N uptake*	101 ± 6.3 aA	130 ± 5.2 aB	89.3 ± 4.4 aA	134 ± 7.8 aB	85.6 ± 4.3 aA	137 ± 4.0 aB	87.7 ± 1.7 aA	164 ± 4.7 bB
	NUE		19.2 a		29.9 b		34.1 b		51.1 c
Mean^‡^	Grain yield *	5.95 ± 0.03 aA	6.88 ± 0.11 aB	6.28 ± 0.20 abA	7.56 ± 0.18 bB	5.91 ± 0.09 aA	7.25 ± 0.04 abB	6.37 ± 0.08 bA	7.57 ± 0.23 bB
	NUE		18.1 a		25.5 b		29.9 b		36.8 b

^†^The data shown are means ± standard errors (n = 3); Definitions of the treatment codes are referred to the footnotes of Table 1 and the text. ^‡^Mean values of the 2012 and 2013 growing seasons. *Variable within each row followed by the same lowercase letter are not significantly different among the rice cultivation practices under each N application rate at the P < 0.05 level, and those followed by the same capital letter are not significantly different between the unfertilized and fertilized treatments under each rice cultivation practice at the P < 0.05 level.
