# Supplementary material for: Improving rice production sustainability by reducing water demand and greenhouse gas emissions with biodegradable films
Source: Sci Rep. 2017 Jan 5;7:39855. doi: 10.1038/srep39855 (PMC5214061; doi:10.1038/srep39855)
Supplement: Supplementary Information [file srep39855-s1.pdf]

Supplementary Information

Manuscript title:

Improving rice production sustainability by reducing water demand and greenhouse gas emissions with biodegradable films

Authors:

Zhisheng Yao[1,4,*], Xunhua Zheng[1,2], Chunyan Liu[1], Shan Lin[3], Qiang Zuo[3], Klaus Butterbach-Bahl[4]

[1] State Key Laboratory of Atmospheric Boundary Layer Physics and Atmospheric Chemistry, Institute of Atmospheric Physics, Chinese Academy of Sciences, Beijing 100029, P.R. China

[2] College of Earth Science, University of Chinese Academy of Sciences, Beijing 100049, P.R. China

[3] College of Resource and Environmental Science, China Agricultural University, Beijing 100193, P.R. China

[4] Institute for Meteorology and Climate Research, Atmospheric Environmental Research, Karlsruhe Institute of Technology, D-82467 Garmisch-Partenkirchen, Germany

* Corresponding author

Name: Zhisheng Yao

Tel: 0086-10-62025885

Fax: 0086-10-62041393

E-mail: zhishengyao@mail.iap.ac.cn

Supplementary Information consists of 22 pages, 3 tables and 8 figures.

Auxiliary measurements

The daily precipitation and average air temperature during the two paddy rice-fallow rotation cycles obtained from an on-site automatic meteorological station (WeatherHawk 500, Campbell Scientific, USA) from May 2012 to April 2014 are shown in Figure S1. During the rice-growing periods, the amounts of irrigation water applied to each plot were monitored using a water flowmeter. Furthermore, in each plot, the soil redox potentials (Eh) at depths of 10 cm were measured using platinum-tipped electrodes and a calomel reference electrode connected to a portable millivolt meter (FJA-5, Nanjing Chuan-Di Instrument Co. Ltd., China). The volumetric water content at a soil depth of 0-6 cm was monitored in each GCRPS plot by using a positioned MPM-160 (RDS Technology Co. Ltd., Nanjing, China). The floodwater depths in each CP plot were measured daily using a vertical ruler. In the fallow season, the volumetric soil water content in the 0-6 cm layer was monitored daily in all plots by using a portable frequency domain reflectometry probe. During the entire paddy rice-fallow periods, the soil temperatures at a depth of 5 cm were automatically recorded in both GCRPS and CP plots in 15 min intervals using HOBO temperature sensors. Throughout the observation period, the topsoil ammonium (NH_4^+) and nitrate (NO_3^-) concentrations at a depth of 0-10 cm were measured weekly by analyzing pooled soil samples from all of the plots of a given treatment. Soil samples were collected using a 3 cm diameter gauge auger. The resulting composite sample was separated into three subsamples of 20 g each. The soil samples were placed in 100 ml 1 M KCl to extract NH_4^+ and NO_3^-. Following 60 min of shaking, the supernatant solutions were filtered and analyzed colorimetrically for NH_4^+ and NO_3^-

by using a continuous flow analyzer (San++, Skalar Analytical B.V., Netherlands).

To determine rice yields, 10 m^2 at the center of each plot was harvested at maturity. Harvested rice plants were divided into grain and straw and oven-dried to a constant weight at 70 °C. Subsamples of grain and straw were ground using a professional grinder and subsequently analyzed for their nitrogen concentrations. (1)

During the rice-growing periods of 2012 and 2013, the total rainfall was 630 and 639 mm, respectively, accounting for 82% and 74% of the total annual rainfall, respectively. In addition, the annual mean air temperature was 14.2 and 15.1 °C, respectively, for the 2012-2013 and 2013-2014 cropping years. The mean air temperature during both rice-growing seasons was the same (23.0 °C) (Figure S1). The soil temperatures under all rice cultivation practices showed comparable fluctuations with the air temperature, with a minimum temperature in January/February and a maximum temperature in July. However, the soil temperature, especially during the rice-growing season, was influenced by different cultivation practices (Figures S2a-b, S3a-b, S4a-b and S5a-b). Within the first month after transplanting rice, the average soil temperatures were 21.6, 25.1, 23.8 and 25.3 °C for CP, $GCRPS_{sat}$, $GCRPS_{bio}$ and $GCRPS_{low}$, respectively, indicating that GCRPSs significantly increased the soil temperature by at least 2.2 °C. Among the GCRPS treatments, the polyethylene film (i.e., $GCRPS_{sat}$ and $GCRPS_{low}$) generally resulted in the highest soil temperature when compared with the plots covered by biodegradable film (i.e., $GCRPS_{bio}$). During the fallow periods, the average soil temperatures were 9.8, 10.4, 10.3 and 9.9 °C for CP, $GCRPS_{sat}$, $GCRPS_{bio}$ and $GCRPS_{low}$, respectively, with no significant differences among the treatments.

The floodwater height in the CP plots averaged 2.2 and 3.4 cm for the 2012 and 2013 rice-growing seasons, respectively, except for the periods of midseason drainage and

final drainage (Figure S2c-d). For the GCRPS plots, the soil water content, which was expressed as WFPS (water-filled pore space), ranged from 42% to 96% across the two rice-growing seasons, with mean values of 85% and 82% for $GCRPS_{sat}$, 83% and 80% for $GCRPS_{bio}$ and 73% and 70% for $GCRPS_{low}$ in the 2012 and 2013 growing seasons, respectively. As expected, the $GCRPS_{sat}$ and $GCRPS_{bio}$ treatments had comparable WFPS values that were higher than the WFPS values observed for $GCRPS_{low}$ (Figures S3c-d, S4c-d and S5c-d). During the fallow periods, soil moisture variations were driven by rainfall events, with WFPS values ranging from 43% to 80%. Similar to soil temperature, no significant treatment effects on soil WFPS were observed during the fallow periods, indicating that legacy management effects on both parameters do not exist.

During the rice-growing seasons, the amplitude of soil Eh under each rice cultivation practice (fluctuating between -101 to 400 mV) was primarily affected by soil drying and wetting conditions, which were regulated by water regimes and rainfall events (Figures S2e-f, S3e-f, S4e-f and S5e-f). Averaged across the two rice-growing seasons, the soil Eh was significantly higher in the GCRPS (88-210 mV) treatments than in the CP (27 mV). When compared with $GCRPS_{low}$ (210 mV), the $GCRPS_{sat}$ (88 mV) and $GCRPS_{bio}$ (92 mV) treatments had lower average soil Eh values.

Across the rice-growing seasons, soil NH_4^+ was the dominant mineral form of N in all treatments, with substantial peaks following fertilizer application (Figures S2g-j, S3g-j, S4g-j and S5g-j). However, soil NH_4^+ in the urea-fertilized GCRPS plots were on average 42% higher (mean: 18.0-24.5 mg N kg^{-1}SDW (soil dry weight)) than those of the urea-fertilized CP plots (14.6 mg N kg^{-1}SDW). Additionally, the soil NO_3^- concentrations in the GCRPS treatments were 77% higher, on average, than those in the CP, although their concentrations were generally < 5.0 mg N kg^{-1}SDW. Across the

fallow periods, neither NH_4^+ nor NO_3^- concentrations were significantly influenced by the preceding rice cultivation practices; however, the application of fertilizer during the growing seasons demonstrated a residual influence on soil mineral N, which led to higher NH_4^+ and NO_3^- concentrations in the +N plots compared to the -N plots.

Data processing

Cumulative CH_4 and N_2O fluxes during the rice-growing season, winter fallow period and entire year were estimated using successive linear interpolation of the gas fluxes on the sampling days and by assuming that CH_4 and N_2O fluxes were linear between measurement days. The cumulative CH_4 and N_2O fluxes during the different measurement periods were converted to CO_2 equivalents (CO_2-eq) based on their respective radiative forcing potentials relative to CO_2 (CH_4:34; N_2O: 298) over a 100-yr period (IPCC, 2013). To assess the total and agronomic GHG mitigation potentials of the tested management options, the CO_2 equivalents of seasonal and annual fluxes of CH_4, N_2O and CH_4+N_2O were expressed based on area and yield scales. The direct emission factor (EF_d) for N_2O emissions was defined as the percentage loss of the applied nitrogen fertilizer via N_2O-N emissions in the current year or season, which was calculated as the difference in emissions between the fertilized and unfertilized plots. In the present study, the irrigation water use efficiency (IWUE) was defined as the grain yield divided by the amount of irrigation water supplied for each rice cultivation practice. The fertilizer N-use efficiency (NUE) was computed based on the percentage of the differences in the amount of aboveground N uptake between fertilized and unfertilized plots compared to the fertilizer N input.

Cost-benefit analysis

To further evaluate whether GCRPSs are an economically feasible approach for

reducing environmental impacts and increasing rice yields, we performed a cost-benefit analysis based on ecosystem services following the study of Compton et al. (2) The overall impacts of GCRPSs on ecosystem services were quantified as monetary values, which were determined by summarizing the costs/benefits associated with the impacts of GCRPSs on GHG emissions, irrigation water demands and crop productivity, as well as the expenses of purchasing plastic film and hiring labor for preparing and mulching the fields.

The cost-benefit analysis indicated that the conversion from CP to GCRPS resulted in an average net environmental benefit of \$55.2-75.7 ha^{-1} yr^{-1} across the 2-year study, mainly due to the mitigating effects of the GCRPS treatments on CH_4 emissions (Table S3). In comparison with the CP, the increases in rice yield and decreased demands for irrigation water in the GCRPS resulted in a revenue increase of \$164.0-297.8 ha^{-1} yr^{-1} (Table S3). Although the GCRPS practices require more labor-time than the CP and require the manufacture and purchase of mulching materials, the $GCRPS_{sat}$ and $GCRPS_{low}$ practices have net monetary benefits of \$203.9 and \$251.5 ha^{-1} yr^{-1}, respectively. The benefit for $GCRPS_{bio}$ reaches up to \$39.8 ha^{-1} yr^{-1}, which is lower than the benefits of the $GCRPS_{sat}$ or $GCRPS_{low}$ treatments because the price of the $GCRPS_{bio}$ mulching material is approximately twice the price of the polyethylene plastic film.

References

1. Tao, Y.Y. *et al*. More rice with less water-evaluation of yield and resource use efficiency in ground cover rice production system with transplanting. *Eur. J. Agron.* **68**, 13-21 (2015).
2. Compton, J.E. *et al*. Campbell, H.V. Ecosystem services altered by human changes in the nitrogen cycle: a new perspective for US decision making. *Ecol.*

Lett. **14**, 804-815 (2011).

3. Song, S. Ship emissions inventory, social cost and eco-efficiency in Shanghai Yangshan port. *Atmos. Environ.* **82**, 288-297 (2014).
4. Linquist, B.A. *et al*. Reducing greenhouse gas emissions, water use, and grain arsenic levels in rice systems. *Global Change Biol.* **21**, 407-417 (2015).
5. Gerngross, T.U. Can biotechnology move us toward a sustainable society?. *Nat. Biotechnol*. **17**, 541-544.

Table S1. Soil properties of the rice-fallow system in the study site

Soil depth (cm)	Soil organic carbon (g C kg^{-1}) †	Total nitrogen (g N kg^{-1}) †	Bulk density (g cm^{-3}) †	Particle fraction (%) †		
				2-0.05 mm	0.05-0.002 mm	<0.002 mm
0-10	11.9±0.6	1.31±0.07	1.36±0.04	20.2±0.8	60.0±0.7	19.8±0.3
10-40	9.6±1.1	1.13±0.15	1.53±0.01	16.7±2.1	65.5±0.9	17.8±1.4
40-80	8.1±0.3	1.08±0.04	1.52±0.07	18.6±0.5	64.5±0.5	16.9±0.8

† Data shown are means ± standard errors (n=9)

Table S2. Seasonal emissions of methane (CH_4, in kg C ha^{-1}) and nitrous oxide (N_2O, in kg N ha^{-1}) from the raised bed and plant-free furrow in each ground cover rice production system (GCRPS) fertilized using two nitrogen application rates during the rice-growing seasons of 2012 and 2013

Rice season	Variable	$GCRPS_{sat}$†		$GCRPS_{bio}$†		$GCRPS_{low}$†	
		-N	+N	-N	+N	-N	+N
2012	CH_4_raised bed	27.7 ± 5.9	41.1 ± 3.6	24.6 ± 1.6	24.7 ± 12.8	28.8 ± 11.4	17.0 ± 7.6
	CH_4_furrow	9.41 ± 3.39	6.70 ± 1.78	5.30 ± 0.66	3.49 ± 1.01	4.27 ± 1.22	2.69 ± 0.92
	Area weighted CH_4*	25.3 ± 4.8 aA	36.6 ± 3.2 aA	22.1 ± 1.4 aA	21.9 ± 11.2abA	25.6 ± 10.0 aA	15.1 ± 6.7 bA
	N_2O_raised bed	0.24 ± 0.03	1.66 ± 0.22	0.30 ± 0.07	1.87 ± 0.34	0.42 ± 0.05	2.74 ± 0.77
	N_2O_furrow	0.04 ± 0.03	0.66 ± 0.18	0.07 ± 0.01	0.83 ± 0.42	0.08 ± 0.01	0.87 ± 0.53
	Area weighted N_2O*	0.22 ± 0.02 aA	1.53 ± 0.17 aB	0.27 ± 0.06aA	1.74 ± 0.29abB	0.38 ±0.04 bA	2.49 ± 0.69 bB
2013	CH_4_raised bed	29.6 ± 4.7	31.8 ± 7.0	30.4 ± 9.1	18.3 ± 5.8	15.5 ± 10.8	8.54 ± 1.73
	CH_4_furrow	4.01 ± 1.92	3.90 ± 1.75	2.18 ± 1.47	2.47 ± 1.38	1.73 ± 1.36	2.11 ± 1.29
	Area weighted CH_4*	26.3 ± 4.3 aA	28.1 ± 6.3 aA	26.7 ± 7.7 aA	16.3 ± 4.9 bA	13.7 ± 9.6 bA	7.71 ± 1.45 bA
	N_2O_raised bed	0.18 ± 0.03	3.16 ± 0.16	0.12 ± 0.02	3.77 ± 0.50	0.28 ± 0.02	3.68 ± 0.28
	N_2O_furrow	0.10 ± 0.04	1.92 ± 0.34	0.09 ± 0.03	2.01 ± 0.42	0.06 ± 0.01	2.03 ± 0.48
	Area weighted N_2O*	0.17 ± 0.03 aA	3.00 ± 0.17 aB	0.11 ± 0.02 aA	3.54 ± 0.49 aB	0.25 ± 0.02 bA	3.46 ± 0.23 aB

† The data shown are means ± standard errors (n=3); $GCRPS_{sat}$, the ground cover rice production system with polyethylene films, where soil water content was kept nearly saturated; $GCRPS_{bio}$, the ground cover rice production system with biodegradable films, where water was managed the same as in the $GCRPS_{sat}$ treatment; $GCRPS_{low}$, the ground cover rice production system with the same covering film as the $GCRPS_{sat}$ and with near saturation until the rice-regreening stage and at approximately 80% of the $GCRPS_{sat}$ management for the reminder of the season; -N, no synthetic nitrogen fertilizer application; +N, a local common application rate of 150 kg N ha^{-1}. * The area weighted CH_4 and N_2O emissions were calculated based on the areal extent of the raised bed (87%) and furrow (13%); these emissions within each row followed

by the same lowercase letter are not significantly different among the rice cultivation practices under each N application rate at the $P < 0.05$ level, and those followed by the same capital letter are not significantly different between unfertilized and fertilized treatments under each rice cultivation practice at the $P < 0.05$ level.

Table S3. Averaging the values from the investigated years of 2012-2014, the changes in the cost/benefit analysis of the ground cover rice production systems (GCRPSs) treated with a nitrogen application rate of 150 kg N ha^{-1} yr^{-1} relative to the conventional paddy (CP) were determined. For the changes of the variables (CH_4 and N_2O emissions, plastic film, labor demand, irrigation water and rice production) under GCRPSs, positive values indicate that the GCRPS practices increased their value and negative values indicate that the GCRPS practices reduced their value. For the monetary response, positive values indicate the amount of economic benefit and negative values indicate the economic cost

Variables	Assessed impacts	Cost/Unit price (data source)	Changes in variables under GCRPS			Monetary response ($ ha^{-1} yr^{-1})		
			$GCRPS_{sat}$	$GCRPS_{bio}$	$GCRPS_{low}$	$GCRPS_{sat}$	$GCRPS_{bio}$	$GCRPS_{low}$
CH_4 emission	The cost of climate change	$1.08 kg^{-1} C (3)	-51.8 kg C ha^{-1}	-65.1 kg C ha^{-1}	-72.6 kg C ha^{-1}	55.9	70.4	78.4
N_2O emission	The cost of climate change	$2.17 kg^{-1} N (2)	0.31 kg N ha^{-1}	-0.07 kg N ha^{-1}	1.24 kg N ha^{-1}	-0.67	0.16	-2.69
Plastic film	The cost of purchasing film	$3.23 kg^{-1} for biodegradable film*, $1.77 kg^{-1} for polyethylene film*	50 kg ha^{-1}#	50 kg ha^{-1}#	50 kg ha^{-1}#	-88.7	-161.3	-88.7
Film manufacture	Estimate of the CO_2 use to produce the film	$0.029 kg^{-1} CO_2 (3)	113 kg CO_2 ha^{-1}‡	120 kg CO_2 ha^{-1}†	113 kg CO_2 ha^{-1}‡	-3.3	-3.5	-3.3
Labor	The cost of time-intensive field preparation and mulching	$10.0 ha^{-1} $labor^{-1}$ &	3 labors&	3 labors&	3 labors&	-30.0	-30.0	-30.0
Irrigation water	The benefit of saving water	$0.00996 m^{-3}§ (4)	-4090 m^3 ha^{-1}	-3930 m^3 ha^{-1}	-6340 m^3 ha^{-1}	40.7	39.1	63.1
Rice production	The benefit of increasing yield	$0.337 kg^{-1} (4)	683 kg ha^{-1}	370 kg ha^{-1}	697 kg ha^{-1}	230.0	124.9	234.7
Sum of the monetary responses						203.9	39.8	251.5

* The prices of the plastic film represent the mean local market price of the biodegradable foil Ecoflex® or regular polyethylene film. # The amount of plastic film application was determined according to agronomist recommendations and a practical farm survey. ‡ The amount of CO_2

emission was calculated by multiplying the application amount of polyethylene films (i.e., 50 kg ha^{-1}) and an estimate of 2.26 kg CO_2 per kilogram of polyethylene film during the manufacture process (5). † The amount of CO_2 emission was calculated by multiplying the application amount of biodegradable films (i.e., 50 kg ha^{-1}) and an estimate of 2.39 kg CO_2 per kilogram of biodegradable film during the manufacture process (5). & The price and amount of labor demand were determined from interviews with local farmers. § The price of irrigation water was calculated by multiplying the amount of diesel for raising 1 m^3 of water from the well (ca. 012 L m^{-3}) and a diesel cost of $0.83 per liter, which was estimated by Linquist et al. (4)

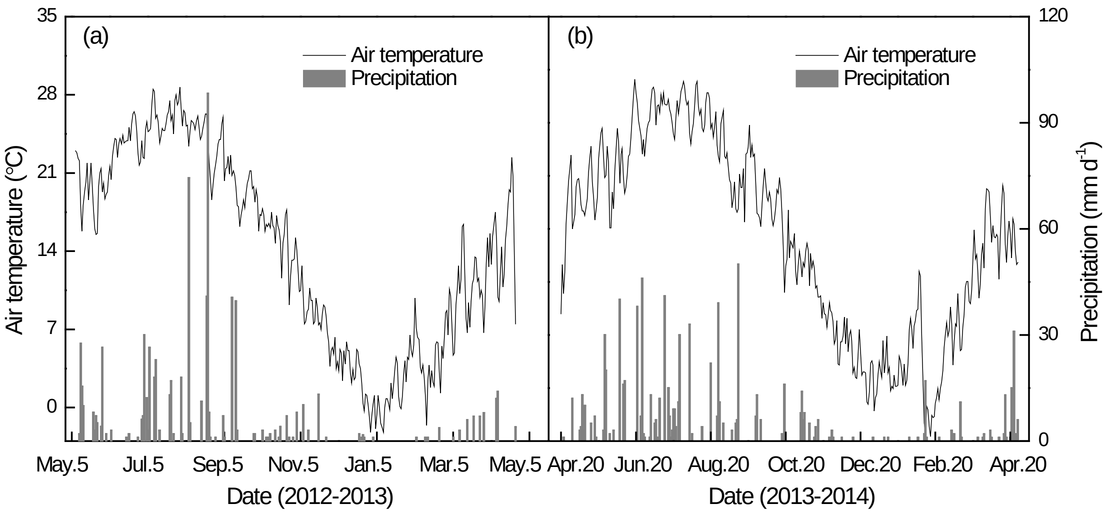

Figure S1. Temporal variations of (a-b) air temperature and daily precipitation for the 2012-2013 and 2013-2014 cropping years.

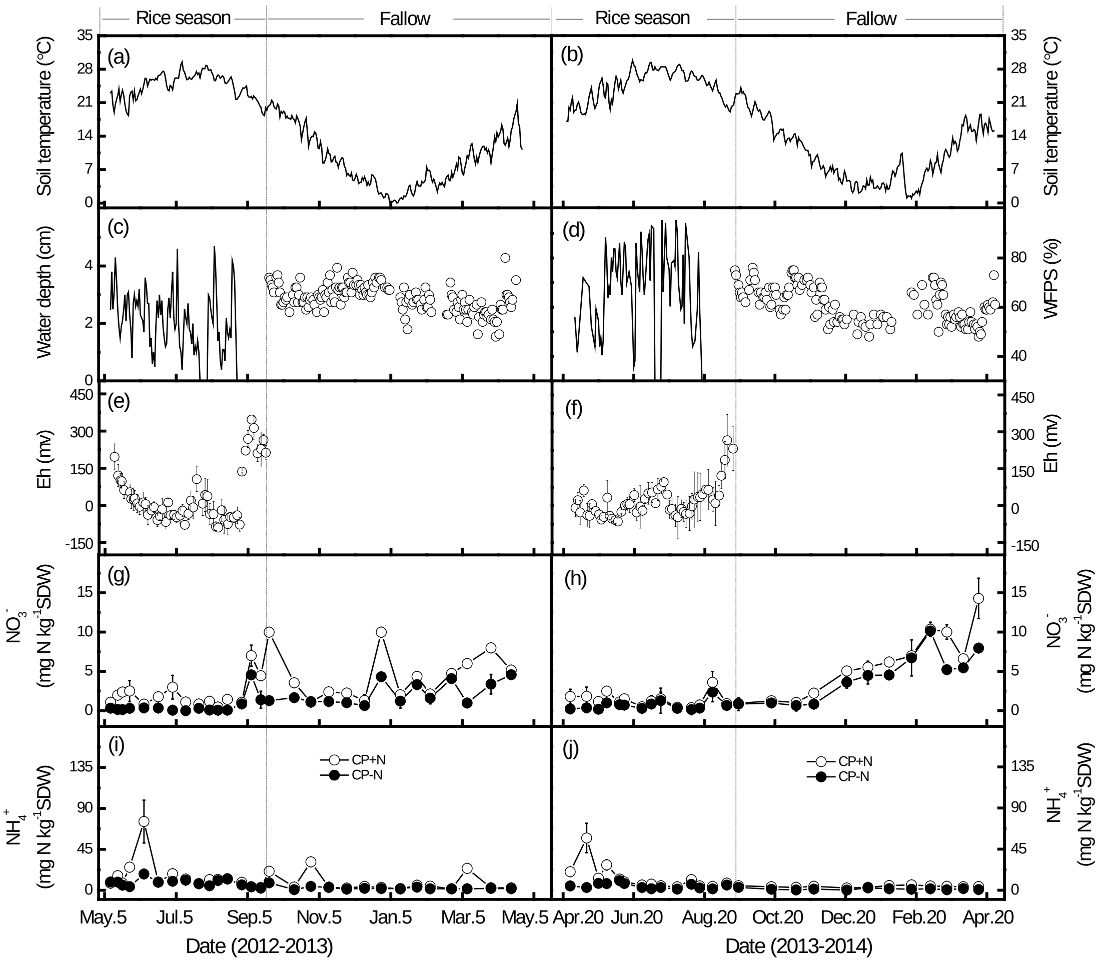

Figure S2. Seasonal variations of (a-b) soil temperature at a depth of 5 cm, (c-d) floodwater depth during the growing season and WFPS (water-filled pore space) at a soil depth of 0-6 cm during the fallow season, (e-f) redox potential (Eh) at a soil depth of 10 cm, soil (g-h) nitrate (NO_3^-) and (i-j) ammonium (NH_4^+) concentrations for the conventional paddy (CP) fertilized using two nitrogen application rates (-N, no nitrogen addition; +N, urea application at a common rate of 150 kg N ha^{-1}) in each annual rice-fallow system of 2012-2014. Vertical bars indicate the standard errors of three replicates. SDW stands for the soil dry weight.

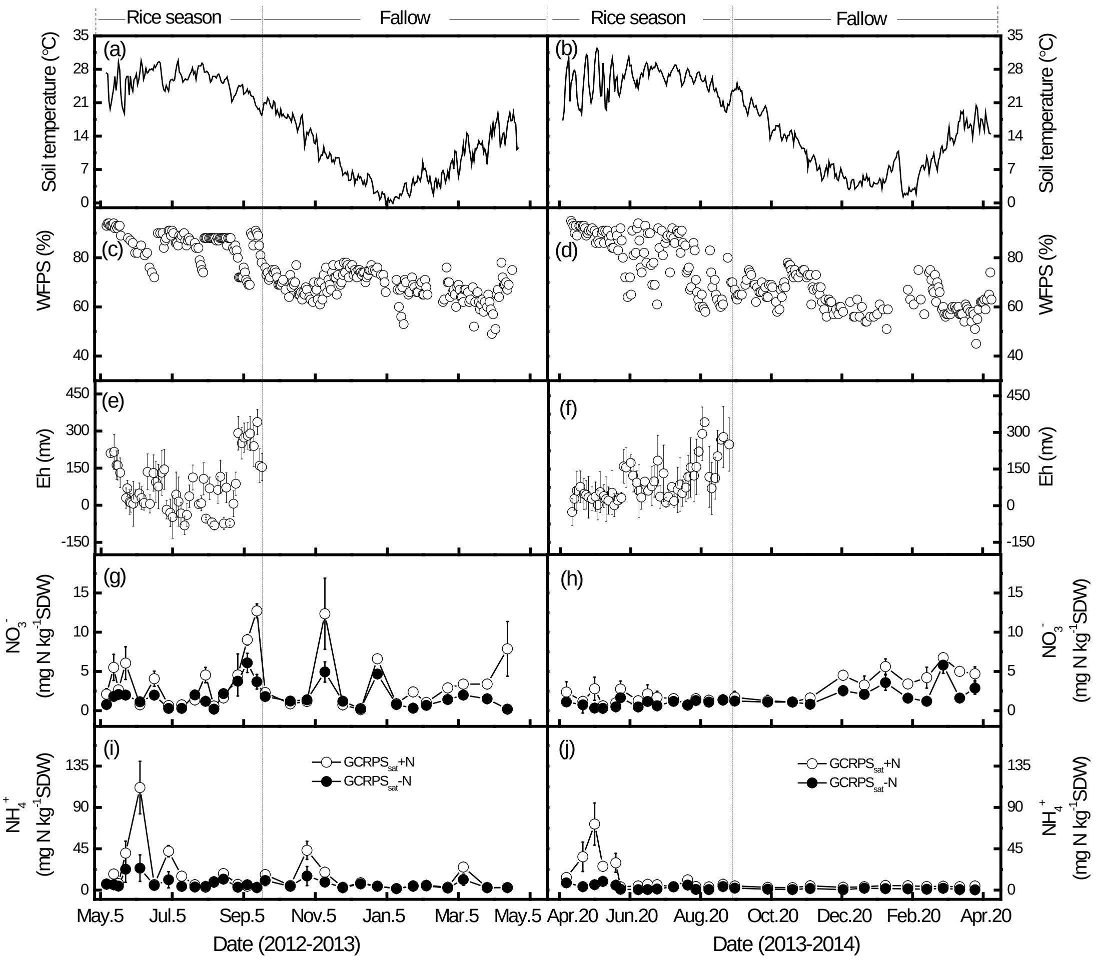

Figure S3. Seasonal variations of (a-b) soil temperature at a depth of 5 cm, (c-d) WFPS (water-filled pore space) at a soil depth of 0-6 cm, (e-f) redox potential (Eh) at a soil depth of 10 cm, soil (g-h) nitrate (NO_3^-) and (i-j) ammonium (NH_4^+) concentrations for the ground cover rice production system under nearly saturated soil conditions ($GCRPS_{sat}$) fertilized using two nitrogen application rates (-N, no nitrogen addition; +N, urea application at a common rate of 150 kg N ha^{-1}) in each annual rice-fallow system of 2012-2014. Vertical bars indicate the standard errors of three replicates. SDW stands for the soil dry weight.

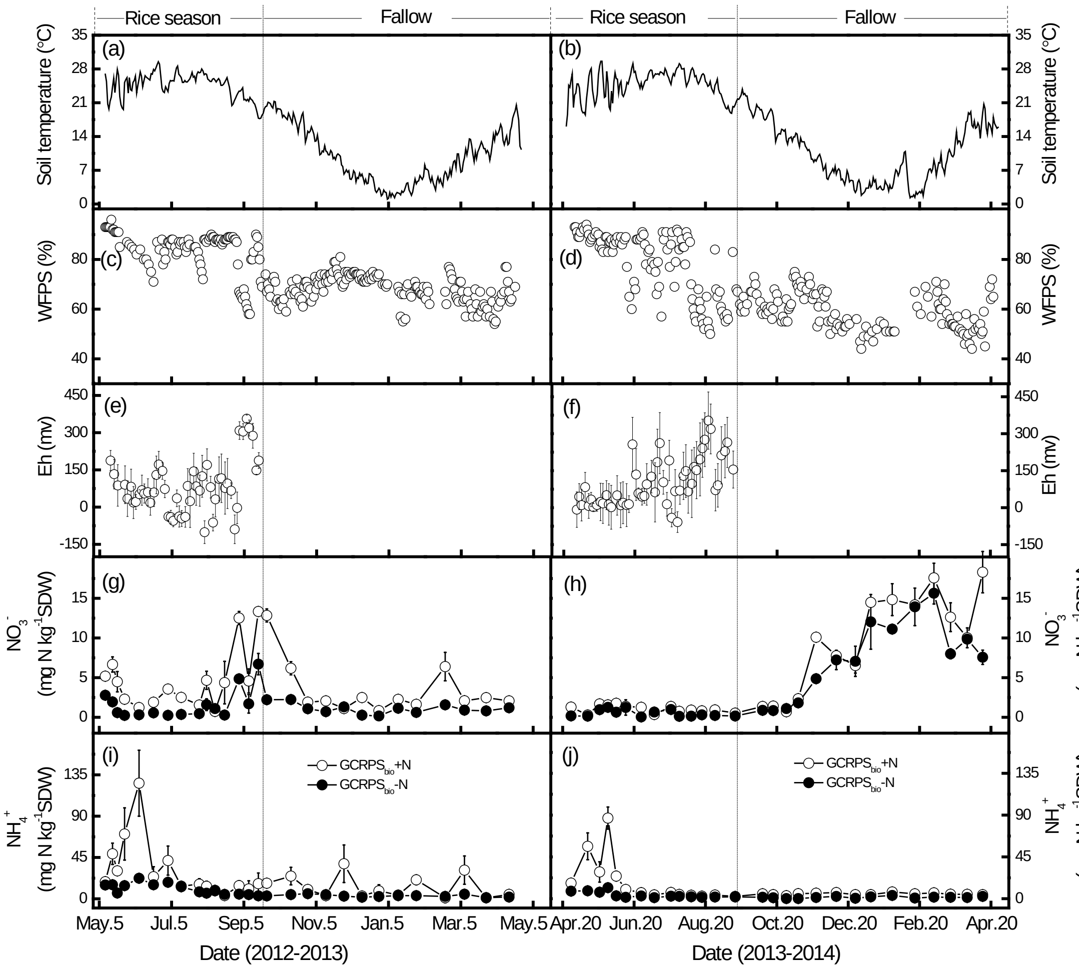

Figure S4. Seasonal variations of (a-b) soil temperature at a depth of 5 cm, (c-d) WFPS (water-filled pore space) at a soil depth of 0-6 cm, (e-f) redox potential (Eh) at a soil depth of 10 cm, soil (g-h) nitrate (NO_3^-) and (i-j) ammonium (NH_4^+) concentrations for the ground cover rice production system using biodegradable films ($GCRPS_{bio}$) fertilized using two nitrogen application rates (-N, no nitrogen addition; +N, urea application at a common rate of 150 kg N ha^{-1}) in each annual rice-fallow system of 2012-2014. Vertical bars indicate the standard errors of three replicates. SDW stands for the soil dry weight.

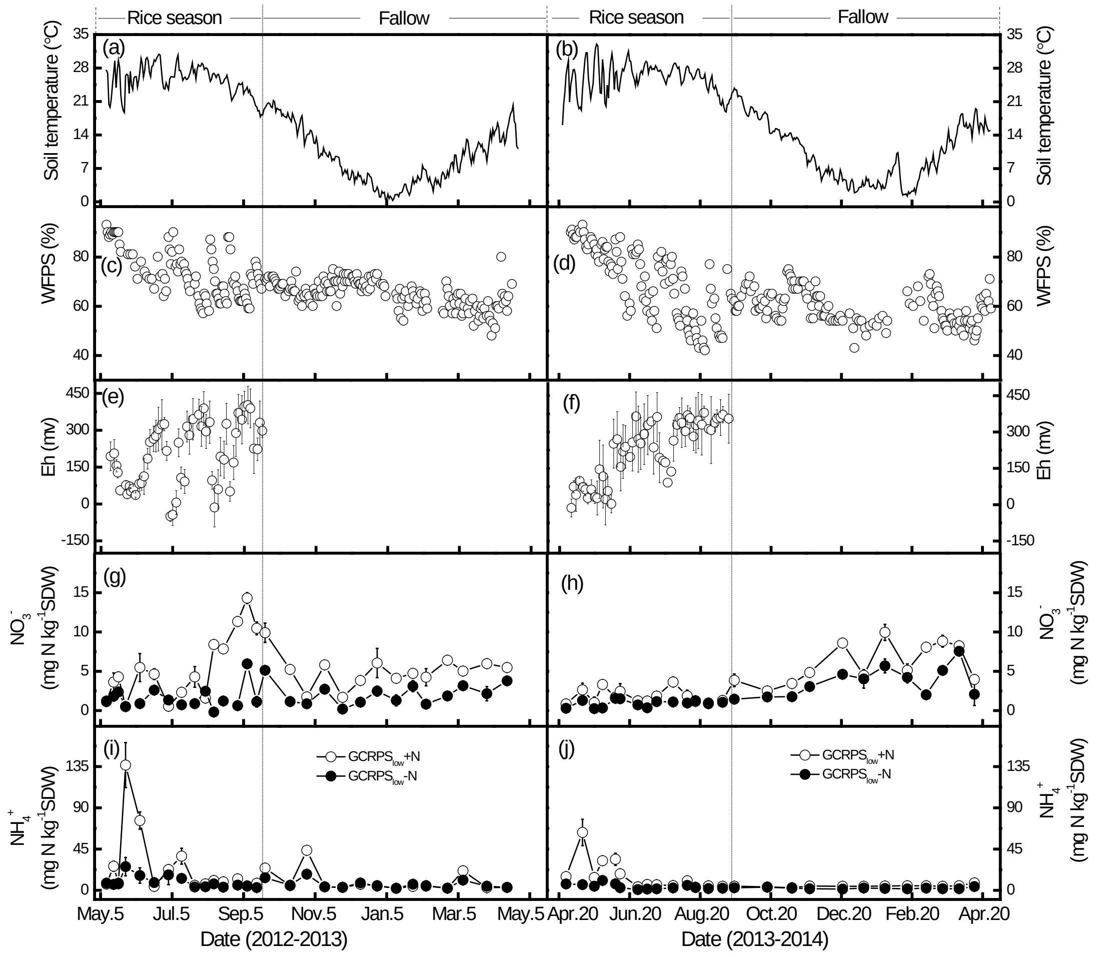

Figure S5. Seasonal variations of (a-b) soil temperature at a depth of 5 cm, (c-d) WFPS (water-filled pore space) at a soil depth of 0-6 cm, (e-f) redox potential (Eh) at a soil depth of 10 cm, soil (g-h) nitrate (NO_3^-) and (i-j) ammonium (NH_4^+) concentrations for the ground cover rice production system with lower soil water statuses (i.e., keeping near saturation until the rice-regreening stage and at unsaturated soil conditions for the reminder of the season, GCRPS$_{low}$) fertilized using two nitrogen application rates (-N, no nitrogen addition; +N, urea application at a common rate of 150 kg N ha^{-1}) in each annual rice-fallow system of 2012-2014. Vertical bars indicate the standard errors of three replicates. SDW stands for the soil dry weight.

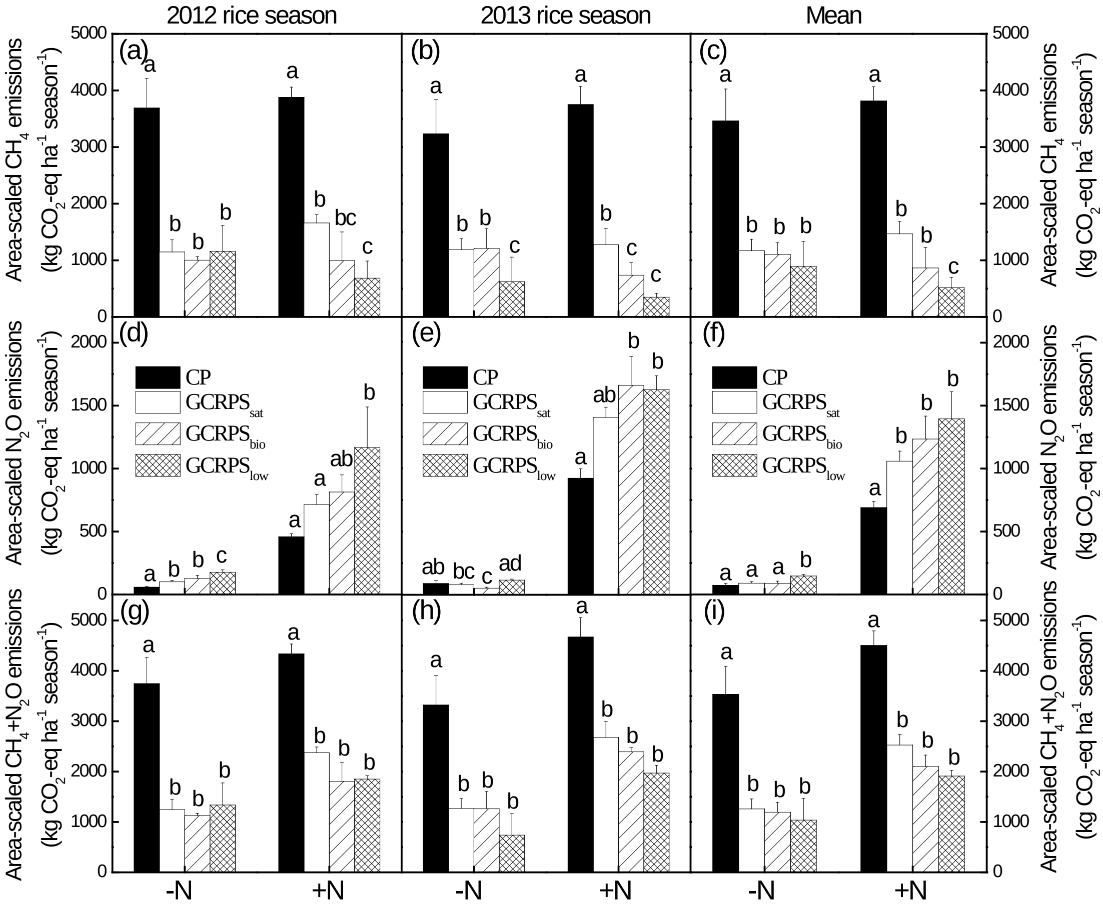

Figure S6. Area-scaled carbon dioxide (CO_2) equivalents of methane (CH_4), nitrous oxide (N_2O) and CH_4 plus N_2O emissions for different rice cultivation practices fertilized using two nitrogen application rates (-N, no nitrogen addition; +N, urea application at a common rate of 150 kg N ha^{-1}) during the rice-growing seasons of 2012 and 2013. Mean represents the mean values of the two growing seasons. Vertical bars indicate the standard errors of three replicates in each rice cultivation practice. The area-scaled CO_2 equivalents of CH_4, N_2O and CH_4+N_2O emissions for each N application rate followed by same letter are not significant at $P < 0.05$. CP, the conventional paddy rice production system with an initial flooding-midseason drainage-reflooding irrigation mode; $GCRPS_{sat}$, the ground cover rice production system with polyethylene films when the soil water content was kept nearly saturated; $GCRPS_{bio}$, the ground cover rice production system with biodegradable films, where

water is managed the same as in the $GCRPS_{sat}$ treatment; $GCRPS_{low}$, the ground cover rice production system with the same covering film as $GCRPS_{sat}$ and the soil water content maintained near saturation until the rice-regreening stage and at approximately 80% of the $GCRPS_{sat}$ management for the reminder of the season.

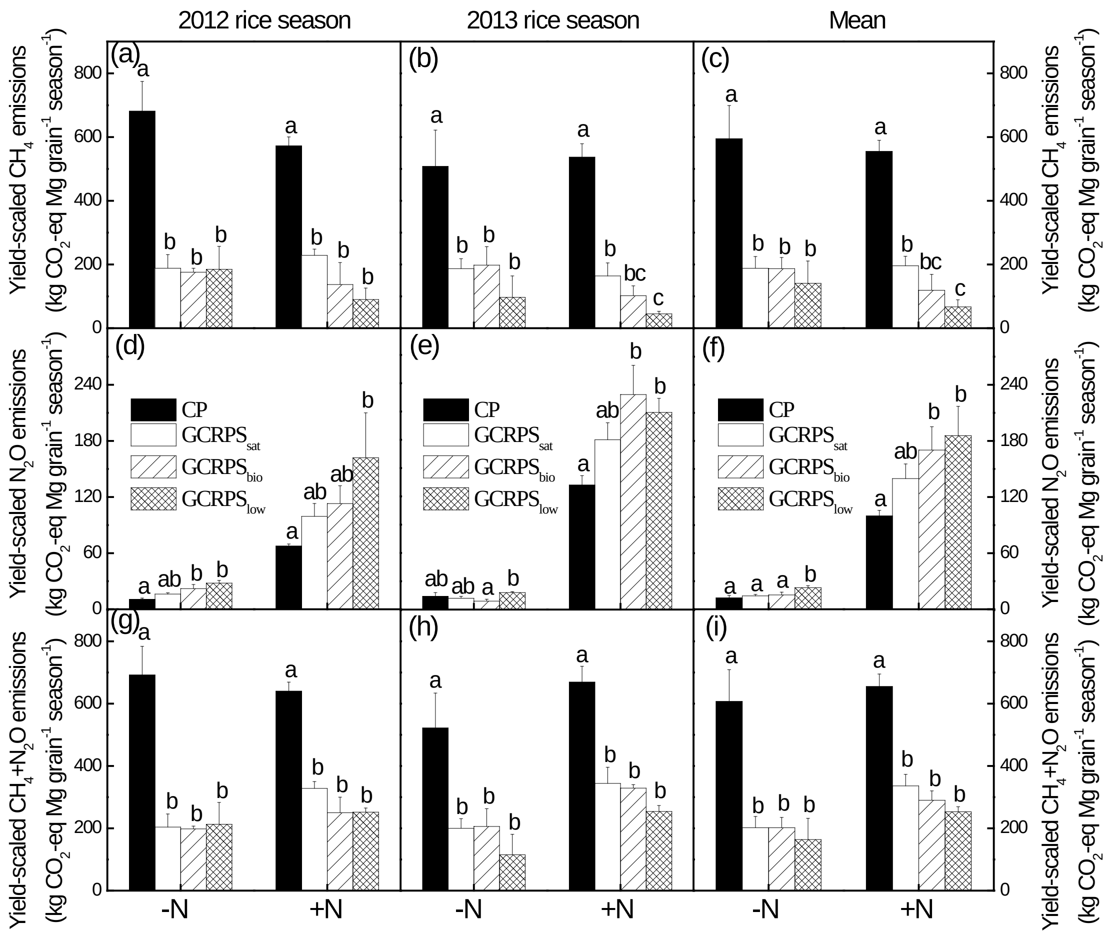

Figure S7. Yield-scaled carbon dioxide (CO_2) equivalents of methane (CH_4), nitrous oxide (N_2O) and CH_4 plus N_2O emissions for different rice cultivation practices fertilized using two nitrogen application rates (-N, no nitrogen addition; +N, urea application at a common rate of 150 kg N ha^{-1}) during the rice-growing seasons of 2012 and 2013. Mean represents the mean values of the two growing seasons. Vertical bars indicate the standard errors of three replicates in each rice cultivation practice. The yield-scaled CO_2 equivalents of CH_4, N_2O and CH_4+N_2O emissions for each N application rate followed by same letter are not significant at $P < 0.05$. CP, the conventional paddy rice production system with an initial flooding-midseason drainage-reflooding irrigation mode; $GCRPS_{sat}$, the ground cover rice production system with polyethylene films when the soil water content was kept nearly saturated; $GCRPS_{bio}$, the ground cover rice production system with biodegradable films, where water is managed the same as in the $GCRPS_{sat}$ treatment; $GCRPS_{low}$, the ground cover

rice production system with the same covering film as $GCRPS_{sat}$ and the soil water content maintained near saturation until the rice-regreening stage and at approximately 80% of the $GCRPS_{sat}$ management for the reminder of the season.

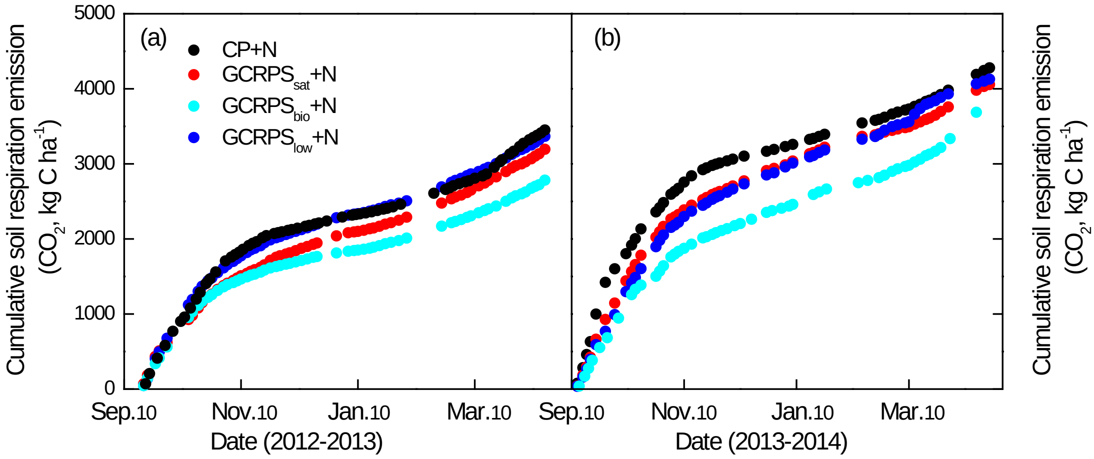

Figure S8. Seasonal cumulative emissions of soil respiration (CO_2) from the different rice cultivation practices fertilized with urea application at a common rate of 150 kg N ha^{-1} (+N) during the fallow season of 2012-2013 (a) and 2013-2014 (b). CP, the conventional paddy rice production system with an initial flooding-midseason drainage-reflooding irrigation mode; $GCRPS_{sat}$, the ground cover rice production system with polyethylene films when the soil water content was kept nearly saturated; $GCRPS_{bio}$, the ground cover rice production system with biodegradable films, where water is managed the same as in the $GCRPS_{sat}$ treatment; $GCRPS_{low}$, the ground cover rice production system with the same covering film as $GCRPS_{sat}$ and the soil water content maintained near saturation until the rice-regreening stage and at approximately 80% of the $GCRPS_{sat}$ management for the reminder of the season.
